# A Miniature Gas Sampling Interface with Open Microfluidic Channels: Characterization of Gas-to-Liquid Extraction Efficiency of Volatile Organic Compounds

**DOI:** 10.3390/mi10070486

**Published:** 2019-07-19

**Authors:** Andrew C. Warden, Stephen C. Trowell, Murat Gel

**Affiliations:** 1 CSIRO Land and Water, Canberra, ACT 2601, Australia; 2 CSIRO Health and Biosecurity, Canberra, ACT 2601, Australia; 3 CSIRO Manufacturing, Clayton, VIC 3168, Australia

**Keywords:** gas-to-liquid extraction, VOC, open channel, microfluidic, capillary flow

## Abstract

Chemosensory protein based olfactory biosensors are expected to play a significant role in next-generation volatile organic compound (VOC) detection systems due to their ultra-high sensitivity and selectivity. As these biosensors can perform most efficiently in aqueous environments, the detection systems need to incorporate a gas sampling interface for gas-to-liquid extraction. This interface should extract the VOCs from the gas phase with high efficiency and transfer them into the liquid containing biosensors to enable subsequent detection. To design such a transfer interface, an understanding of the key parameters influencing the gas-to-liquid extraction efficiency of target VOCs is crucial. This paper reports a gas sampling interface system based on a microfluidic open-channel device for gas-to-liquid extraction. By using this device as a model platform, the key parameters dictating the VOC extraction efficiency were identified. When loaded with 30 μL of capture liquid, the microfluidic device generates a gas-liquid interface area of 3 cm^2^ without using an interfacial membrane. The pumpless operation based on capillary flow was demonstrated for capture liquid loading and collection. Gas samples spiked with lipophilic model volatiles (hexanal and allyl methyl sulfide) were used for characterization of the VOC extraction efficiency. Decreasing the sampling temperature to 15 °C had a significant impact on increasing capture efficiency, while variation in the gas sampling flow rate had no significant impact in the range between 40–120 mL min^−1^. This study found more than a 10-fold increase in capture efficiency by chemical modification of the capture liquid with alpha-cyclodextrin. The highest capture efficiency of 30% was demonstrated with gas samples spiked with hexanal to a concentration of 16 ppm (molar proportion). The approach in this study should be useful for further optimisation of miniaturised gas-to-liquid extraction systems and contribute to the design of chemosensory protein-based VOC detection systems.

## 1. Introduction

The detection of volatile organic compounds (VOCs) has application in a wide range of industries including food, agriculture, health and security. While conventional detection technologies such as semiconductor based solid state e-nose systems [1,2] are progressing, there is growing interest in employing chemosensory protein-based biosensors available in olfactory systems in nature as the recognition elements with a view to exploit their superior detection sensitivity and selectivity [3,4,5,6]. Towards the ultimate goal of developing next-generation VOC detection systems, the use of whole-cell assays [7], odorant binding proteins [8], olfactory receptors [9,10], peptides [11] and aptamers [12] have been proposed. One of the key challenges in using these biosensors in VOC-detection systems is the requirement for an efficient gas sampling interface to transfer the VOCs from the gas phase into the liquid phase. This is because the biosensors are made of proteins which are functional only in aqueous solutions. This problem is further complicated by the physicochemical properties of certain volatile compounds, which are of significant interest in the health and security domains. The properties, such as lipophilicity and low water solubility, limit the transfer efficiency of the compounds into an aqueous solution, therefore reducing the overall sensitivity of the VOC detection system. To use biosensors as part of field-deployable the VOC detection systems, a well-characterised gas-to-liquid transfer strategy needs to be established and the key factors influencing VOC capture efficiency need to be identified to enable system optimization.

A number of studies examining gas-to-liquid transfer methods for the detection of the VOCs by biosensors have been published. Park et al. [13] used a porous polycarbonate membrane separating a gas flow chamber and olfactory cells to develop a platform for mimicking the human olfactory system. Stemme et al. [14] used a microfabricated perforated silicon membrane to establish a gas-liquid interface for subsequent detection using antibodies. Toda et al. [15] used a polydimethyl siloxane (PDMS) membrane to construct a micro scrubber. However, mass transfer through membranes is a limiting factor for detection speed and collection efficiency [16]. Furthermore, there is a risk of adsorption of the VOCs on the membrane material, causing contamination and sample carry over.

Miniature systems based on open channels that do not rely on any membrane structure have also been reported [17,18]. These systems provide an effective way to establish a large gas-liquid interface area and eliminate issues related with membranes. These interfaces used surface wettability to spread a small volume of liquid over an area. However, in these studies, maintaining the gas-liquid interface required complex pumping systems for interface stabilization which prevents the interface from drying and the liquid phase from flooding over the open channel structure. The requirement for pumping systems increases the overall complexity and makes it difficult to deploy the technology for industrial applications. There is a desire to develop simpler liquid-handling strategies for utilising gas-liquid interfaces in open channels. The volatile capture efficiency of these interfaces has never been systematically studied, especially for the case of lipophilic molecules with low water solubility.

This study reports a gas-sampling interface based on gas-to-liquid extraction using an open capillary channel device and this study further demonstrates capillary flow-driven loading and collection of capture liquid without using pumps. The pumpless operation provides simplicity by eliminating the requirement for any external active pressure control system, making it more desirable for field deployable applications. By using this device as a model system, the key factors influencing VOC extraction efficiency for lipophilic VOCs were identified. The impact of parameters such as sampling temperature, gas flow rate and chemical modification of the capture liquid composition have been studied.

### 1.1. Device Structure and Operation

**Figure 1:**
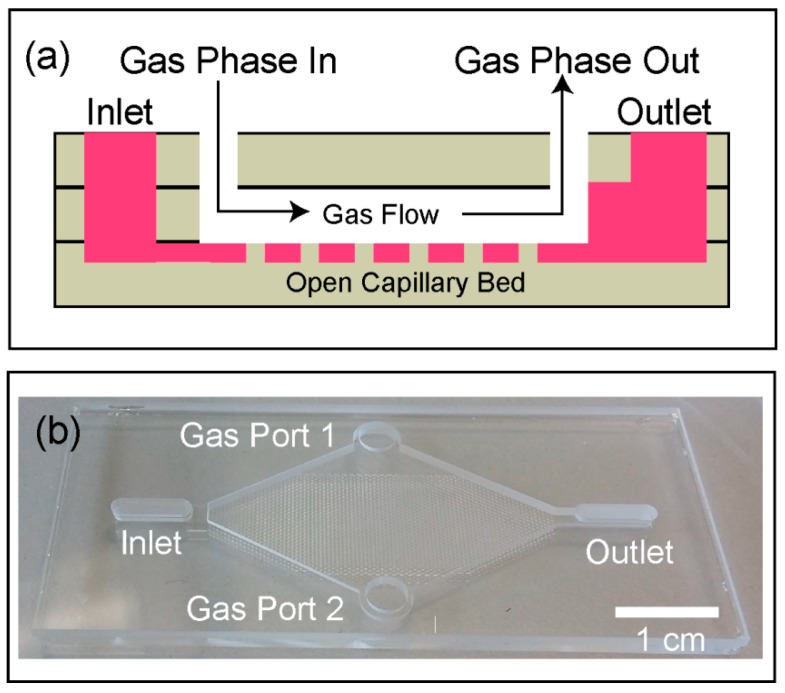
(**a**) The structure of the open-channel device for gas sampling, and (**b**) a photograph of the fabricated all-glass gas-sampling device.

**Figure 2:**
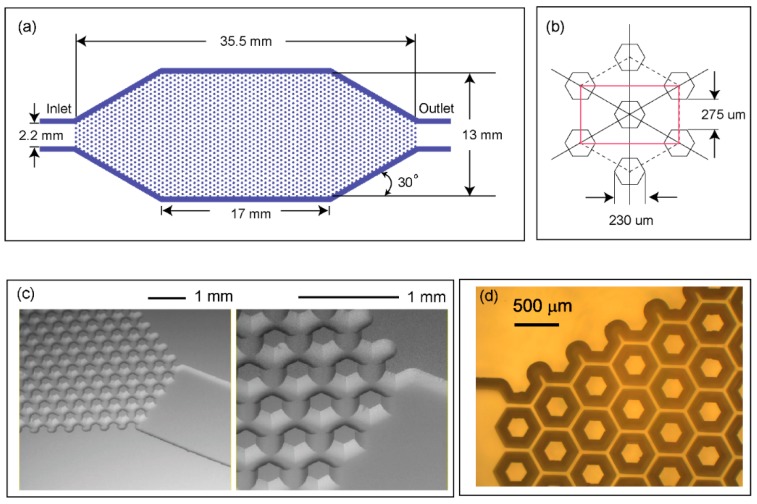
(**a**) A schematic of the open-channel capillary network, (**b**) dimensions of the repeating unit cell (red rectangle), (**c**) SEM image of the fabricated open-channel, and (**d**) optical microscope top view image of the open-channel.

A schematic of our microfabricated, open-channel device is shown in Figure 1a, with an image of a test device shown in Figure 1b. The device has external dimensions of 65 × 30 × 3.3 mm and is composed of three layers of bonded borosilicate glass. The device includes an inlet and outlet for the liquid phase and two circular ports (4 mm diameter) for introducing the gas sample. The device is designed such that the gas sample can be passed through the device across the liquid surface in the open-channel network at a controlled flow rate. The volume of the gas chamber is approximately 500 µL and the open channel network holds 30 µL of capture liquid. The gas-liquid interface area is approximately 3 cm^2^. For the initial priming, the liquid is introduced into the device through the liquid inlet on the left side. The liquid spreads throughout the open channel network by capillary action. The details of the open channel network design are shown in Figure 2a. The design involves hexagonal protrusions between which the capture liquid sits. Figure 2b shows the dimensions of the repeating unit cell. A scanning electron microscope (SEM) image of the capillary bed is shown in Figure 2c. An optical microscope view of the device is shown in Figure 2d. Gas sampling is achieved by introducing the gas sample through the device via the gas ports. During this process, the VOCs in the gas phase are transferred into the liquid in the open channel. The capture liquid is collected from the device for analysis via the outlet with a gas tight syringe (see Section 2.6 for details of collection procedure).

### 1.2. Computational Modelling

**Figure 3:**
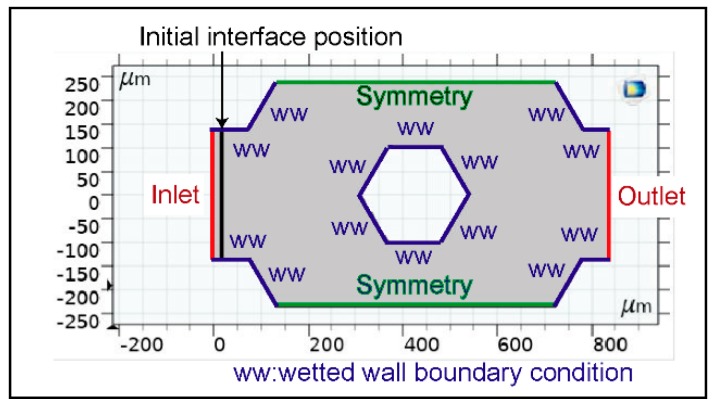
The developed model for flow analysis and the boundary conditions.

The use of capillary flow significantly simplifies device operation by eliminating the requirement for an external pumping system. Capillary flow depends on the surface wettability dictated by contact angle. Computational modelling was used to analyse the contact angle dependence of the capillary flow. COMSOL Multiphysics (www.comsol.com) with the level set method was used to model two-phase flow and track movement of the air-water interface. To minimize computation time, a simplified 2D analysis of the repeating unit cell illustrated in Figure 2b was used as the model geometry. Figure 3 shows the details of the model geometry. The inlet and outlet boundary conditions were set to zero pressure for spontaneous flow condition. The symmetry boundary condition was selected for the top and bottom walls, effectively modelling an infinite 2D expansion of the repeating pattern. The wetted wall boundary conditions in the Multiphysics interface provide user set contact angles. The contact angle range between 5 and 60 degrees was studied.

Using the fluid dynamics interface in COMSOL, the model is solved with two sets of governing equations. The level set method uses Equation (1) to solve the moving interface, where φ is the level set function which is equal to zero in one fluid domain (water) and equal to one in the other (air). Across the interface, there is a continuous transition from zero to one. The velocity field of the interface is represented by **u**. The parameter ε determines the thickness of the region where φ goes smoothly from zero to one and is set to the same order as the size of the mesh elements. γ is the re-initialization parameter, which is set to the maximum magnitude of the velocity field **u**.

(1) $$\frac{\partial \emptyset }{\partial t}+u\cdot \nabla \emptyset =\gamma \nabla \cdot \left(\varepsilon \nabla \emptyset -\emptyset \left(1-\emptyset \right)\frac{\nabla \emptyset }{\left|\nabla \emptyset \right|}\right)$$

The other set of equations solved in the model are continuity and Navier-Stokes Equations (2) and (3), which describe the mass and momentum transport for fluids. The surface tension is included in the model to account for capillary effects. *ρ* denotes the density, *μ* equals the dynamic viscosity, **u** represents velocity, and *p* denotes pressure. *F_st_* is the surface tension acting at the air-water interface.

(2) ρ∇·(u)=0

(3) $$\frac{\partial u}{\partial t}+\rho \left(u\cdot \nabla \right)u=\nabla \cdot \left[-pI+\mu \left(\nabla u+{\left(\nabla u\right)}^{T}\right)\right]+F_{st}$$

## 2. Materials and Methods

### 2.1. Fabrication

**Figure 4:**
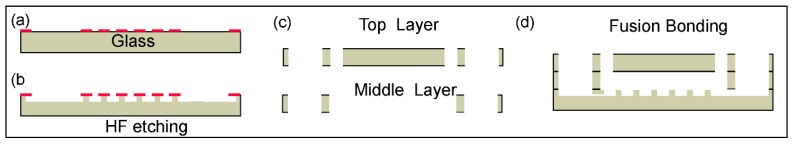
Schematic showing fabrication steps. (**a**) Photolithography and metal patterning, (**b**) open capillary channel layer fabrication with HF etching, (**c**) laser machining of top and middle layer, and (**d**) fusion bonding of the three layers.

The 3-layer device was fabricated from borosilicate glass (1.1 mm thickness) using standard microfabrication techniques. First, a patterned Cr/Au metal layer was fabricated on the glass substrate using photolithography and etching (Figure 4a). The process was followed by hydrofluoric acid (HF), etching to a depth of 100 µm for creating the structures that form the open channel network (Figure 4b). The middle layer and the top layers were fabricated using laser machining (Figure 4c). Finally, the three-layer stack was fusion bonded by annealing at 700 °C (Figure 4d).

### 2.2. Surface Preparation and Maintenance

As the device is based on capillary flow, stable operation relies on maintaining the water wettability of the open channel surface. The wettability of the borosilicate glass surface was achieved by cleaning the surface in aqueous NaOH solution (100 mg·mL^−1^) to generate OH groups on the glass surface. This cleaning procedure was used whenever surface wettability needed to be restored. The fabricated device was immersed in the solution overnight while making sure the surface of the open channel was completely covered by solution without residual bubbles. The device was rinsed with Milli-Q water to wash out remaining NaOH. Surface wettability was confirmed by priming the device with food colouring solution and observing liquid spreading in the open-channel network. For long term storage, the surface was wetted with Milli-Q water and all the ports on the device were sealed with Kapton tape to prevent evaporation and surface drying.

### 2.3. Model Volatiles

The characterization of gas-to-liquid extraction efficiency was performed using gas samples spiked with model volatiles. Table 1 shows the selected volatiles and their properties. The two model volatiles with distinct physicochemical properties were selected to assess the impact of compound lipophilicity on the gas-liquid extraction efficiency. Hexanal and allyl methyl sulfide are also known as breath biomarkers for lung cancer and malaria, respectively.

### 2.4. Gas Sample Preparation

A 50 mL round bottomed glass flask with an outlet and inlet port was heated to 200 °C in a bead bath on a hotplate, well above the boiling points of the volatile compounds. The VOC (3 µL) was injected into the sealed flask through the inlet port to vaporize the compound. The flask was allowed to equilibrate at this temperature for 5 min, after which nitrogen gas was passed through the flask inlet port at a rate of 50 mL min^−1^. The nitrogen line was equipped with a gas flow meter (TSI Inc., Shoreview, MN, USA, SKU: 4146) with a volumetric flow rate display to monitor the total volume of the gas added. The gas sample bag (SKC Inc., Eighty Four, PA, USA, SamplePro FlexFilm sample bag with volumes: 1/3/5 L) was connected to the flask outlet to collect the VOC spiked gas sample. The gas flow was stopped when the capacity of the sample bag was reached. For further dilutions, 100 mL of the initial bag contents was injected into a separate bag and nitrogen was added depending on the dilution level needed. The freshly prepared gas sample bags containing nitrogen and the volatile compound were used for the experiments. The concentrations of the volatiles in the bag were confirmed by sampling 10 mL of gas in a sealed GC vial and analysing with a GC-MS (Agilent 6890 gas chromatograph (GC) coupled with an Agilent 5973 mass selective (MS) detector and automatic headspace sampler (7694E)).

### 2.5. Capture Liquid Solution

The capture solution was 100 mg mL^−1^ alpha-cyclodextrin (Sigma, Aldrich, MI, USA, C4642) in pure Milli-Q water, unless otherwise indicated. The solution was freshly prepared each day.

### 2.6. Device Priming and Capture Liquid Collection

The spontaneous capillary flow was used to prime the chip, and for loading and collection of the capture liquid. The open channel was initially primed with water, then 15 µL of capture liquid was introduced through the inlet after which the water at the outlet was removed by pipette. This procedure was repeated by introducing an additional 15 µL of capture liquid to complete the loading. Once the loading was complete, the inlet and outlet ports were emptied completely by pipette. At this stage, the gas-liquid interface was stabilised by surface tension and the device was ready for gas sampling. To collect a 30 µL capture solution following exposure to the VOC, it was displaced by introducing 2 × 15 µL water at the inlet. This corresponds to approximately 30 µL of the capture liquid sample collected from the device for each measurement. (Supplementary video is available demonstrating capture liquid loading and collection.)

### 2.7. Gas Sampling

**Figure 5:**
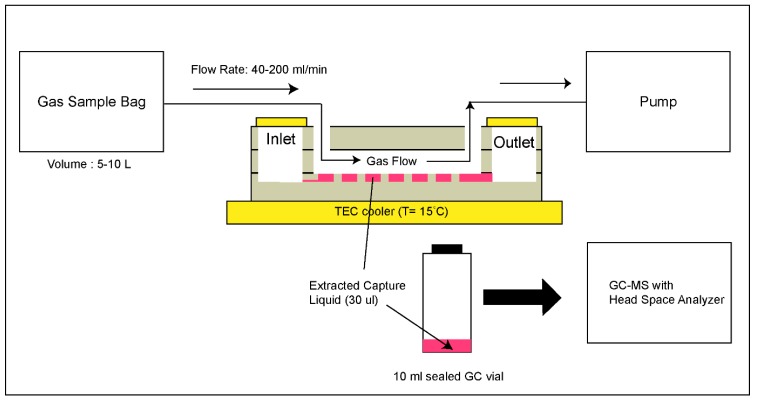
The gas sampling method used for device characterization.

The device was loaded with fresh capture liquid. The inlet and outlet ports for liquid were sealed with Kapton tape. The gas sampling was performed as illustrated in Figure 5. The device was placed on a copper plate attached to a thermo-electric cooler with a control unit (PLC-24V6A, Kurag Electronics, Tokyo, Japan) which allowed temperature adjustment between 15 °C and 40 °C. The diffusion pump oil (Santovac 5, Santovac fluids, Spartanburg, SC, USA) was used between the copper plate and the device to ensure efficient thermal contact. The gas inlet port on the device was connected to the gas sample bag and the gas outlet was connected to a miniature air pump (SKC Inc., 210-1002A). The pump provided negative pressure to the gas outlet of the device to pull the gas from the sample bag in to the device at a controlled flow rate (40–200 mL min^−1^). At the end of gas sampling, the capture liquid (approximately 30 μL) was collected from the device using a gas-tight syringe. The VOC content of the collected liquid was analysed by GC-MS. For analysis, the 30 µL liquid samples were injected into a sealed 10 mL GC vial and immediately placed in the headspace sampler for analysis. For each trace, the peak areas were calculated using Chemstation software (Agilent Technologies, Santa Clara, CA, USA).

### 2.8. Fluoresence Measurements

To quantify the capture liquid collection efficiency, a water soluble fluorescent stain was used as a marker. A calcein (CAS 1461-15-10) stock solution was prepared in water to a concentration of 78 µM, filtered through a 0.22 µm pore size PTFE syringe membrane filter. This solution was loaded to the device and collected to measure the amount of calcein. The plate reader (Enspire 2300 multilabel reader, Perkin Elmer, Waltham, MA, USA) was used to measure fluorescence emission from liquid samples collected from the device. For 96-well plate measurements, the 30 µL collected volume was increased to 100 µL by adding 70 µL of water. The measured emission from the well was converted to calcein amount (moles) by using the calibration curves generated with the standard samples.

## 3. Results

### 3.1. Capillary Flow Simulation

**Figure 6:**
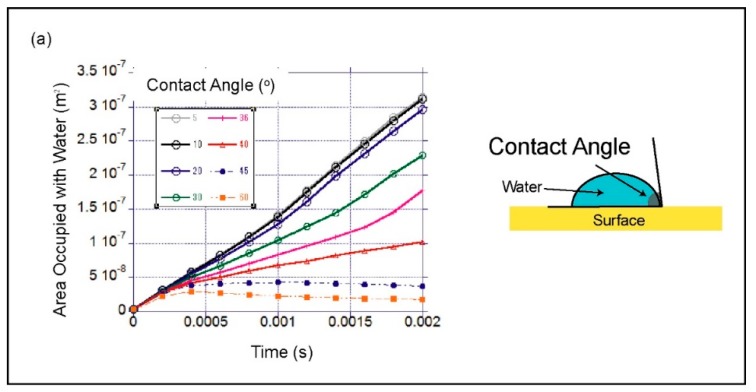

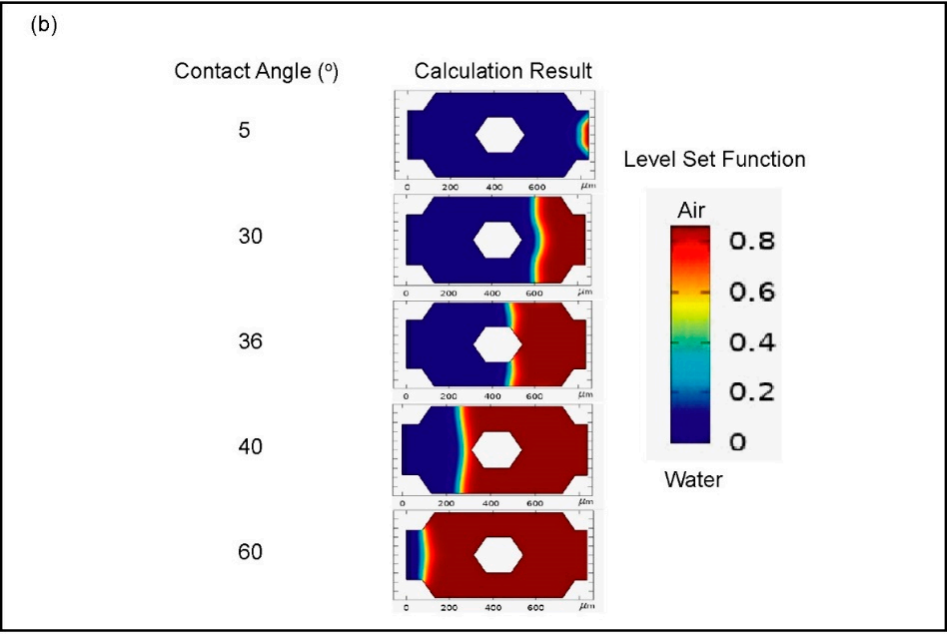
Spontaneous capillary flow simulation with the model for the repeating cell geometry in the open-channel, (**a**) calculated total area occupied with water versus time for contact angles between 5 and 60°, and (**b**) visualization of the simulated repeating cell geometry at 2 ms, with the blue representing the area filled with water and red representing air (flow going from left to right).

The pumpless operation of the device uses capillary flow for priming, capture solution loading and collection. It was found that to enable a stable pumpless operation, the surface wettability and the contact angle of the channel surface must be maintained. To estimate the allowable range of contact angles, this study simulated the capillary flow with contact angles between 5 and 60 degrees. The simulations predicted strong contact angle dependence in the capillary flow behaviour. Figure 6a illustrates the calculated results for the area occupied by water with respect to time. For high wettability conditions with contact angles less than 20 degrees, the liquid velocity during capillary flow was predicted to be approximately 400 mm s^−1^. This calculated velocity was significantly larger than the experimental value for spontaneous flow observed in glass capillaries [19]. The total area occupied by water at the end of 2 ms is illustrated in Figure 6b as a function of the contact angle. For contact angles larger than 40 degrees, the simulation predicted that the air-water interface stalls and spontaneous capillary flow stops. This study observed similar behaviour when the liquid in the open channels dried and contamination was deposited on the surface. Therefore, to obtain a stable operation, the authors ensured the surface remained wet when the device was not in use, to maintain its surface wettability.

### 3.2. Pumpless Operation for Loading and Collection

**Figure 7:**
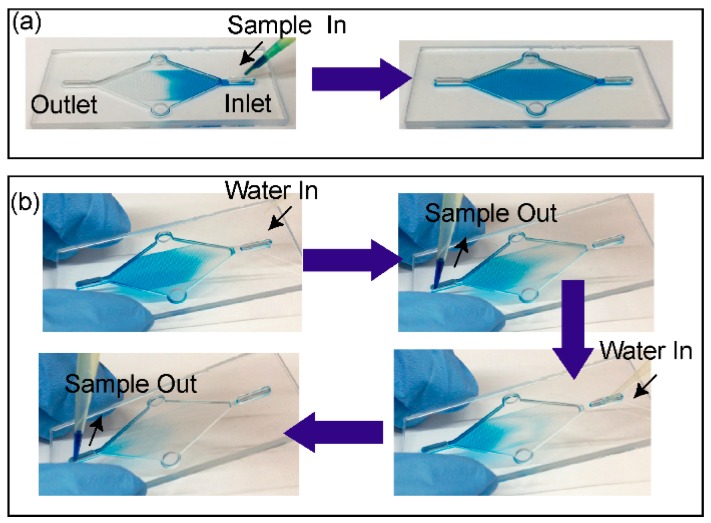
A visualisation of the pumpless device operation with food colouring. The process for (**a**) loading, and (**b**) collection.

The device design enabled the pumpless operation for liquid capture loading and collection. This study visualised the steps using food colouring (the video is available in the supporting material). Figure 7a shows spontaneous spreading when 15 µL of food colouring is introduced through the liquid inlet. The fully loaded chip image was obtained after removing the liquid at the outlet and introducing another 15 µL of food colouring solution. Figure 7b shows the sequence of images during the collection process. When 15 µL of water was introduced, the food colouring moved towards the outlet where it was removed. The collection process was completed by repeating the same sequence one more time.

### 3.3. Quantification of Capture Liquid Collection Efficiency

**Figure 8:**
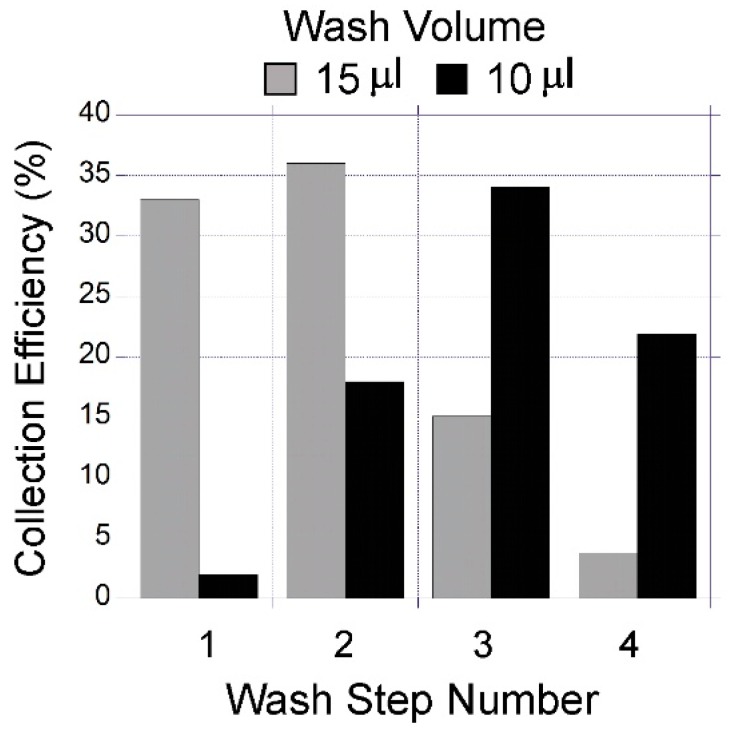
A comparison of the liquid collection efficiency from the device using wash volumes of 10 and 15 µL.

To quantify the capture liquid collection efficiency, the device was loaded with a solution containing a water-soluble fluorescent stain (calcein). After loading, the liquid was collected from the device using the procedure illustrated in Figure 7. The amount of calcein in the collected liquid was quantified by measuring the fluorescence emission from the liquid with a plate reader. Two conditions were tested by using wash volumes of 15 µL and 10 µL. The collection efficiency with respect to each wash step and wash volume used showed varying behaviour (Figure 8). During the wash step, water introduced to the inlet mixes with sample on the device. The mixture emerges from the outlet with a concentration lower than the initial sample. The concentration of the sample in the water varies depending on the wash step number. When higher volumes of wash step were used, the higher concentrations can be eluted earlier than in the case of using lower wash volumes. The peak collection efficiency was 36% with 15 µL wash volumes in the 2nd wash step. In the case of 10 µL wash volumes, the peak collection efficiency of 34% obtained in the 3rd wash step was observed.

To calculate the overall collection efficiency of the system, this study examined the case for four wash steps resulting in a total collected sample volume of 60 µL and 40 µL for wash volumes of 15 µL and 10 µL, respectively. The overall efficiency obtained in the collected total volume can be calculated by summing the efficiencies of each wash step. Accordingly, the overall collection efficiency was calculated as 87.8% and 75.9% for extracted total volumes of 60 µL and 40 µL respectively. To obtain the highest concentration in the collected liquid, two sets of 15 µL wash steps were optimal, generating 30 µL of collected liquid and were used in all the experiments.

### 3.4. Identification of the Factors Influencing the VOC Capture Efficiency

To characterize the volatile capture efficiency of the system, gas samples spiked with model volatile compounds, hexanal and allyl methyl sulfide (AMS) were used. This study defined the capture efficiency as the ratio of the quantity of the captured compound to the input amount. The captured quantity refers to the moles of the VOC measured in the 30 µL of the liquid collected from the device. The input amount corresponds to the moles of the compound input in the device which was calculated by the sample gas concentration and gas volume which passed through the device.

(4) $$\mathrm{Capture}\;\mathrm{Efficiency}=\frac{\mathrm{Captured}\;\mathrm{amount}\;\mathrm{in}\;\mathrm{the}\;\mathrm{liquid}\;\left(moles\right)}{\mathrm{Input}\;\mathrm{amount}\;\left(moles\right)}$$

#### 3.4.1. Dependence of Capture Efficiency on Compound Type, Sampled Gas Amount and Concentration

To characterize the capture efficiency, gas samples spiked with model volatiles at various concentrations were passed through the device. By varying the gas volumes between 50 and 500 mL, the device was exposed to a wider range of input moles of target VOC. The input moles of the VOC that were exposed to the device were calculated as follows:

input moles = gas concentration (nmol/mL) × gas volume passed in to the device (mL)(5)

**Figure 9:**
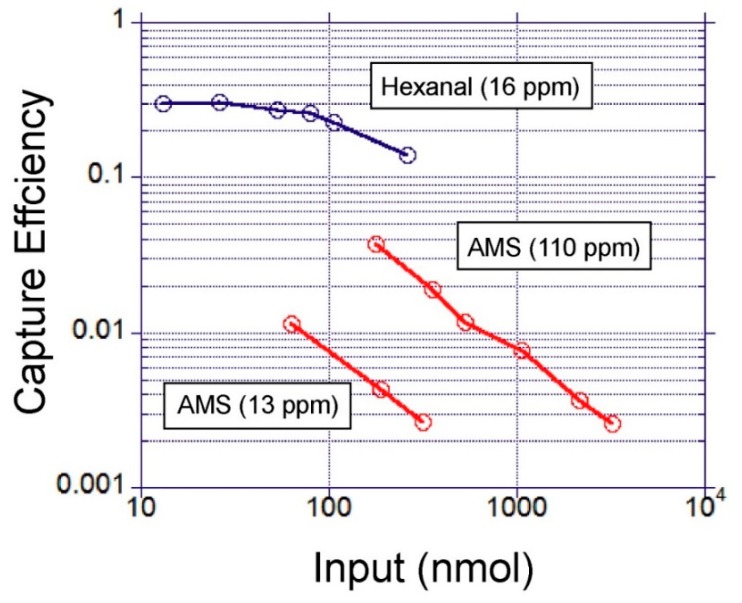
A comparison of the volatile capture efficiency of allyl methyl sulfide (AMS) and hexanal with respect to molar gas input amount to the device (T = 15 °C, Flow rate = 40–100 mL min^−1^). The input amounts were varied by sweeping the volume of the input gas (50–500 mL).

After each experiment, the moles of the captured VOC were measured in the collected liquid. It was observed that the capture efficiency varied significantly depending on the physicochemical properties of the VOC (Figure 9). Significantly higher capture efficiency was obtained with hexanal in comparison to AMS. This result is consistent with the physicochemical properties summarised in Table 1. The higher water solubility of hexanal increases the absorption capacity of water for this VOC. This is compounded with the high vapour pressure of AMS which makes it more volatile in comparison to hexanal and difficult to retain in water. The VOC concentration in the sample gas has also influenced the measured capture efficiency. This can be explained by faster mass transfer rates driven by the steeper concentration gradient. The highest capture efficiency obtained with hexanal was 30% at an input concentration of 16 ppm. For AMS, the highest capture efficiency was 3.7% and 1.1% when the input concentrations were 110 ppm and 13 ppm, respectively. The maximum concentration of the captured hexanal in a 30 µL of collected liquid was 1.2 mM at an input gas concentration of 16 ppm. For AMS, it was 27 µM at an input gas concentration of 13 ppm. The sensitivities (detection limits) of the odorant binding proteins reported in the literature have been in the order of fM [9]. The concentrations obtained with this gas sampling system were at least µM. Therefore, in principal, it should be satisfactory to enable detection with such biosensors. However, the spiked gas samples have much higher concentrations than the levels required for field applications. Therefore, further validation experiments at lower concentrations are needed to demonstrate feasibility in that setting.

#### 3.4.2. The Influence of the Capture Liquid Chemical Modification

**Figure 10:**
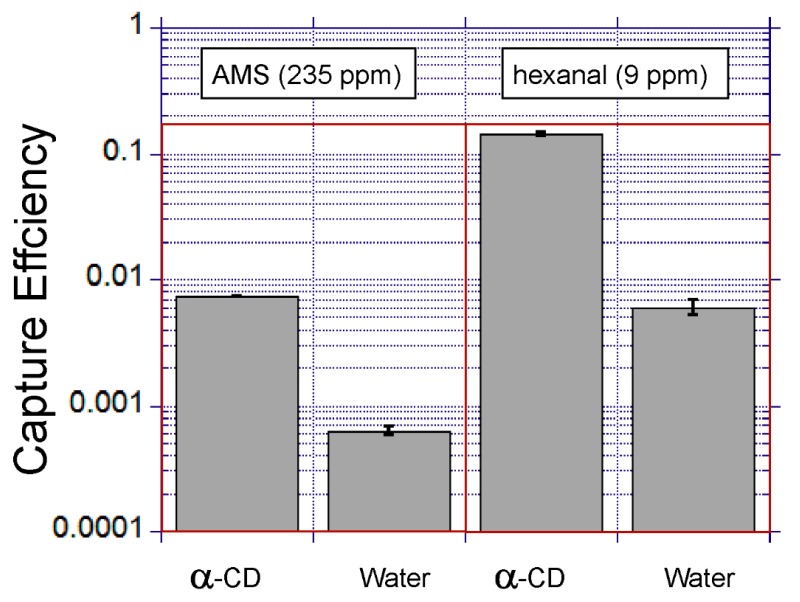
A comparison of the effect of chemical modification of the capture liquid. The error bars show the standard deviation for duplicate experiments (α-CD = alpha-cyclodextrin).

It was found that chemical modification of the capture liquid had a significant impact on the capture efficiency (Figure 10). In particular, the addition of alpha-cyclodextrin into the capture liquid improved the capture efficiency of AMS and hexanal 11.7-fold and 23.6-fold, respectively. Alpha-cyclodextrin has been previously studied as a pseudo-phase, increasing the solubility of lipophilic compounds in aqueous solutions [20]. The authors attribute the increase in capture efficiency to the improved solubilisation of the volatile lipophilic compounds driven by partitioning into the cyclodextrin pseudo-phase.

#### 3.4.3. The Influence of Gas Sampling Temperature and Flow Rate

**Figure 11:**
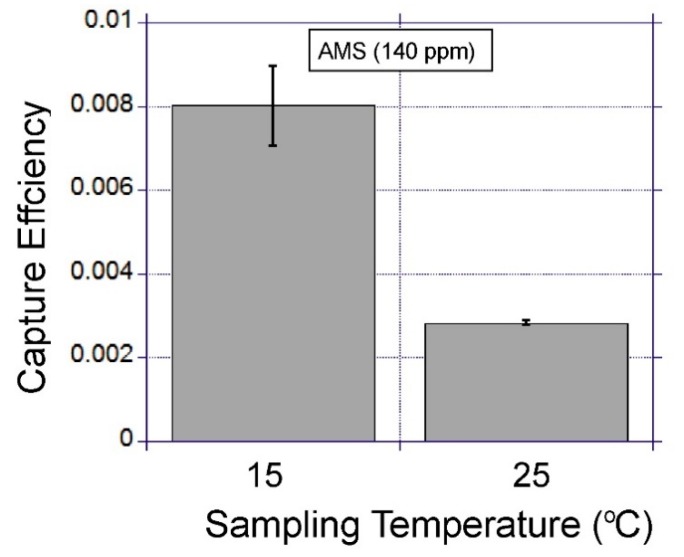
The impact of gas sampling temperature on capture efficiency. The error bars show the standard deviation for duplicate experiments.

**Figure 12:**
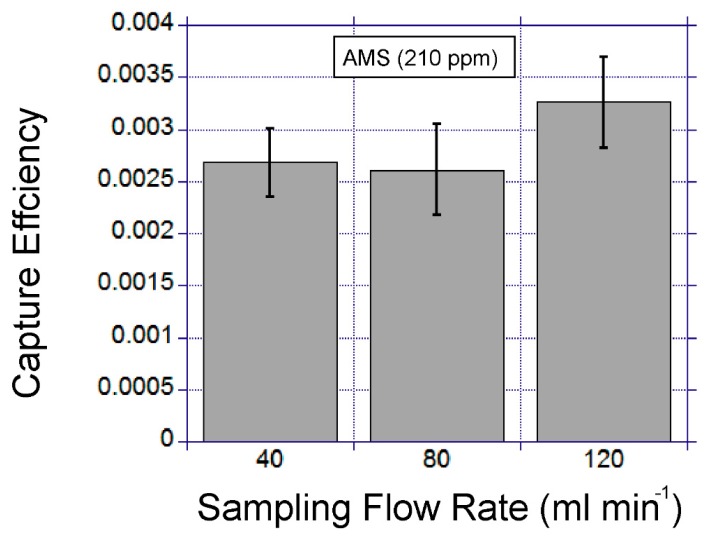
The flow rate influence on capture efficiency. The error bars show the standard deviations for duplicate experiments.

Other physical parameters, such as temperature and flow rate, have been assessed in terms of their impact on the capture efficiency. The device is positioned on a thermo-electric cooler which allows the device temperature to reduce below room temperature. For this measurement AMS-spiked gas samples (400 mL) were passed through the device. This study found that a 10 °C decrease in the device temperature provided a 2.8-fold increase in VOC extraction efficiency (Figure 11). The flow rate had minimal impact on the capture efficiency for the flow rate range between 40 and 120 mL·min^−1^ (Figure 12).

## 4. Conclusions

This study demonstrated the use of an open micro-channel device which can be used as a miniature gas-sampling interface for VOC extraction. The device design utilises capillary flow for capture liquid loading and collection. This is an effective and simple way to charge, sample and recharge a gas-to-liquid extraction device. It was found that maintaining the surface wettability was the key factor in enabling the stable operation of the device. The factors influencing the VOC extraction efficiency were identified using two lipophilic model volatiles with low water solubility. This study showed that gas sampling temperature, chemical modification of the capture liquid and the type of volatile molecule had significant impacts on the capture efficiency. To develop highly efficient gas-to-liquid extraction systems, the identified factors need to be further optimised taking into consideration the properties of target VOCs. This study used GC-MS for VOC detection. However, in the future, the authors envision that detection can be done on a microfluidic system by mixing the extracted liquid sample directly with the biosensors in solution. The proposed gas-sampling interface should be a useful tool to contribute to the development of miniature VOC sensing systems based on biosensors, paving the way for their use in real life applications.

## Figures and Tables

**Table 1:** The physicochemical properties of selected model volatile organic compounds (VOCs).

Compound Name	Water Solubility (g/L at 20 °C)	Vapour Pressure (mmHg at 20 °C)	Boiling Point (°C)
Hexanal	4.8	10	130
Allyl Methyl Sulfide	2.4	52	93

## Supplementary Materials

The following is available online at https://www.mdpi.com/2072-666X/10/7/486/s1, Video S1: Device Priming.
